# Current Atrial Fibrillation Ablation: An Alert for the Prevention and Treatment of Esophageal Lesions

**DOI:** 10.5935/abc.20160078

**Published:** 2016-05

**Authors:** Mauricio Scanavacca

**Affiliations:** Instituto do Coração - HC-FMUSP, São Paulo, SP - Brazil

**Keywords:** Atrial Fibrillation, Catheter Ablation, Arrhythmias, Cardiac, Anti-Arrhythmia Agents, Anticoagulants

Atrial fibrillation (AF) is recognized as the most frequent cardiac arrhythmia in clinical practice, and is responsible for the highest number of hospital admissions. In the general population, its prevalence is lower than 0.1% in individuals younger than 50 years, but increases exponentially with age, affecting about 8% of elderly individuals older than 80 years. Therefore, with the increase in the population's life expectancy, a growing number of patients is or will be exposed to the risks of AF in the coming years.^1,2^

The recent introduction of new anticoagulants has brought considerable advances in the prevention of cerebral embolism, the most severe complication of AF.^3,4^ However, the development of more effective antiarrhythmic drugs failed to show progress during this period. Therefore, a large number of patients still live with the limitations imposed by AF, including decreased quality of life due to symptoms such as palpitations, reduced functional capacity, or manifestations of cardiac insufficiency, in addition to the psychological problems proper of these conditions.^5,6^

In this context, catheter ablation has emerged as the most effective treatment for rhythm control in patients with AF, and has been used in a growing number of patients worldwide over the last decade.^1^ Most studies have confirmed the need to achieve electrical isolation of the pulmonary veins (PV) for a successful procedure since the electrophysiological triggering mechanism of this arrhythmia is located predominantly within these veins.^7^

The technique originally conceived for AF ablation aimed at identifying the muscular fibers within the PV with a circular multipolar catheter, followed by their disconnection from the left atrium (LA) in specific points with the application of radiofrequency (RF) to the internal portion of the veins ostia.^8^ Although the procedure was technically very objective in terms of demonstrating the isolation of the PV, subsequent studies have identified a significant risk of venous stenosis and high recurrence rate during clinical follow-up due to reconnections and supposedly persistent arrhythmogenic foci located outside the isolated area, in the PV antra.^8^

These observations have motivated a change in strategy toward the current technique, which aims at (a broader, extraostial) isolation of the PV antra.^1,8^ For this purpose, mapping systems have been developed and progressively improved. These maps allow a precise three-dimensional virtual construction and real-time visualization of the LA anatomy and its venous drainage. In addition, more effective RF discharging systems have been introduced to achieve transmural atrial lesions (catheters with 8-mm tips, followed by catheters with 3.5-mm irrigated tips and, currently, with a contact sensor). Comparative studies have shown a clear progress in success rates during clinical follow-up and a significant reduction in the risk of PV stenosis.^1,9^ However, these technical changes have created conditions for a new complication, that despite rare, is highly lethal: atrial-esophageal fistula (AEF).^10^

The posterior wall of the LA maintains an intimate anatomical relationship with the anterior esophageal wall.^11^ Therefore, displacement of the ablation lines from the PV ostia to the posterior wall of the LA moves the isolation lines toward the esophagus, whose walls may be thermally damaged by contiguity. This damage, in turn, may evolve to a transmural lesion and mucosal erosion, which eventually progresses to an ulcer (due to gastroesophageal acid reflux) and, more rarely, fistulization to the LA.^12^

The initial symptoms of AEF develop classically between 2 to 4 weeks after the ablation (there are rare late cases between 5 and 6 weeks).^10^ These symptoms appear nonthreatening and include mild retrosternal discomfort, fever, and leukocytosis without an apparent cause. If the process is not diagnosed and interrupted, it evolves rapidly to hematemesis and manifestations of septicemia due to systemic and cerebral septic embolism. At this stage, complete recovery, even after reconstructive surgery of the fistula, is rare; approximately 80% of the patients persist with severe neurological impairments or are unable to survive.^10^

Therefore, clinicians who follow up patients after AF ablation must be aware of these facts and make quick decisions to identify the diagnosis and start treatment.

The occurrence of AEF as a consequence of AF catheter ablation was described for the first time in 2004, soon after the technical changes mentioned above.^13,14^ This occurrence was initially interpreted as a transitory problem that could be prevented with recognition of the anatomical associations of the esophagus with the LA and PV during the procedure and by reducing the RF energy output during ablation of the posterior LA wall close to the esophagus. However, after more than 10 years, the occurrence of AEF persists as one of the most concerning complications of AF ablation, due to its unpredictability and severity.

Currently, AEF post-ablation of AF is described in about 0.1% of the procedures, even those performed in experienced centers, without a convincing demonstration that a specific strategy may prevent it for sure.^1,10^ A recent study gathering experience from eight Brazilian centers with 8,500 performed procedures identified 10 cases of AEF (0.116%), all occurring after the introduction of the technique of circumferential PV ablation. The first two cases occurred in 2004 and 2005 when the risk of AEF was still unknown, yielding an incidence of approximately 1%. The other eight cases occurred between 2008 and 2015, yielding an incidence of 0.1% after introduction of several preventive measures such as monitoring of the position of the esophagus, decrease in RF power for applications in the posterior wall of the LA, esophageal temperature monitoring during the ablation, use of electroanatomic mapping to direct the applications, intracardiac echocardiography, and monitoring of the pressure of the ablation catheter.^15^

The increased number of cases in the last period was probably related to the increased number of procedures performed in our country. However, it is worth pointing out that the improvement of the devices to increase the effectiveness of the ablation and reduce PV reconnections (the most frequent cause of recurrence after ablation) may also increase the rates of esophageal lesions if more effective measures to protect the esophagus are not taken.

The most used method among us to prevent thermal esophageal lesions is esophageal temperature monitoring with power adjustments during RF application in the posterior LA wall.^16^ With irrigated-tip catheters, the usual RF application output is 30 to 40 W during the creation of the anterior lines. When the applications are directed to the posterior lines close to the esophagus (identified by a radiopaque thermometer on X-ray), the operator must reduce the power (to 20 W) and application time (20 sec), and avoid high contact force (10 g). In general, an increase in esophageal temperature determines prompt interruption of the application. However, different centers disagree about the limit of the temperature to interrupt the application, ranging from an increase in 1°C from the initial temperature (our approach) or increase to the limit of 38.5°C or 41°C.^17,18^ Depending on the circumstances, the power is reduced to 15 or 10 W, and the application is repeated when the temperature decreases. Low power, on the one hand, hinders the creation of contiguous transmural lesions in slightly thicker tissues and do not always prevent the increase in esophageal temperature. On the other hand, they often decrease the success rates of the procedure. The rate of esophageal erosion varies between 5 and 40% when upper gastrointestinal endoscopy is systematically performed in the first days after the ablation and depends on the techniques and limits to interrupt the application used during the procedure.^10,12,16-18^

The use of esophageal temperature monitoring has some limitations.^19^ The most used thermometer has a single distal electrode on a linear, inflexible catheter that is moved toward the upper and lower esophageal portions to be positioned as close as possible to the location of the ablation in the posterior LA wall. Due to that, an assigned professional must move the thermometer at each movement of the ablation catheter. Another important limitation is that the absence of a critical elevation in esophageal temperature does not necessarily mean that the esophagus is distant, since due to its anatomical characteristics, it may be compressed (thus, not tubular) and the thermometer may occupy only one part of its lumen (which will be well monitored), failing to measure the actual temperature of the contralateral margin.

In order to overcome these two limitations, another esophageal thermometer has been developed, in which multiple electrodes are distributed along the catheter in a sinusoidal format to occupy the flat surface of the esophagus.^20^ The clinical use of this second system has shown higher sensitivity and readiness to detect increases in esophageal temperature. However, there are no randomized studies comparing the efficacy of both systems or the ability of this device in maintaining the esophagus distended, moving it closer to the LA.^19,20^

Another problem also not yet resolved is how to produce effective and safe lesions in areas of the LA clearly close to the esophagus. Considering that the esophagus is free in the thoracic cavity, the most recent promising proposal, which is under evaluation, is the mobilization of the esophagus with a mechanical system dedicated to this purpose. Once the relationship of the esophagus with the ablation area in the LA is documented, this system moves the esophagus from the area at risk, allowing safe and more effective applications.^21^

There are no defined guidelines for the management of patients with esophageal lesions after AF ablation, just as there are no clinical studies proving the efficacy and safety of the procedures that have been used. In our unit, we regularly use esophageal monitoring with one of the systems mentioned above, and we currently perform in all patients upper gastrointestinal endoscopy 24-72 hours after the ablation, regardless of the elevation in esophageal temperature. Our objective with this approach is to identify the actual incidence of esophageal lesions and establish the circumstances in which the endoscopy is absolutely necessary.

High doses of proton pump inhibitors (omeprazole or pantoprazole, 40 mg twice a day) for 30 days have been recommended to reduce the reflux of acid to the esophagus after ablation, independent of the findings of the esophageal temperature monitoring during the procedure. In patients with esophageal lesions (erythema, erosion, and hematoma), we add sucralfate 2.0 g between meals and recommend a pureed light diet. Patients with evidence of some degree of gastroparesis in the endoscopy or with clinical manifestations also receive bromopride (10 mg three to four times a day) for 30 days. With these measures, most acute lesions disappear on endoscopic evaluations performed 7 to 10 days later. However, there are cases in which the lesions progress and acquire features of an active ulcer. The situation becomes more concerning if the patient presents fever. In this condition, the patient must be admitted, remain fasting, and undergo monitoring with leukogram and biochemical markers of inflammatory response (C-reactive protein and procalcitonin). Chest computed tomography (CT) with oral contrast should be performed to investigate a possible fistula formation. Esophagoscopy should be avoided in this situation due to the risk of gas embolization if a fistula is present.

Classical signs found on CT are the presence of gas images or infiltration of contrast in the mediastinum or pericardium. Patients without evidence of infection and normal CT are maintained in the hospital for monitoring while receiving clinical treatment for the ulcer. Patients with an infectious response but without defined fistulization must start prolonged fasting along with hydration and parenteral nutrition, and receive atropine to reduce salivary secretion and broad-spectrum antibiotic therapy. A gastric surgery team must be called to follow up the patient because an emergency intervention may be required.^22^

The CT or magnetic resonance imaging (MRI) scan is repeated 1 week later to evaluate the progress. In the event of clinical evidence of fistulization (systemic or cerebral embolism, or hematemesis) the patient must be operated on immediately. When the procedure is performed within a few hours from the initial manifestation, a good clinical recovery is expected; otherwise, the rates of death or definitive impairments are high.^22^

New technologies for isolation of the PV have been incorporated into clinical practice. Among these, balloon cryoablation has been the most studied. In an initial experience with first-generation balloons (23 mm), AEF was not reported because the lesions were located in the ostia. But with second generation balloons (mainly 28-mm) and more powerful cooling involving the entire anterior face of the balloon, AEF has been reported^23^, so some groups have already started to monitor the esophageal temperature during use of these balloons. In contrast, there are systems in which the RF is applied simultaneously by multiple electrodes mounted on a catheter with a circular tip positioned around the ostia of the PV. These systems allow the operator to apply the RF in all electrodes simultaneously to shorten the procedure. The occurrence of AEF has been already reported with the use of irrigated electrodes (nMARQ).^24^ There are still no reports of AEF occurring with the other system (PVAC), recently introduced in Brazil, in which the electrodes are not irrigated, and the applications are intermittent. However, endoscopies performed after the ablation have shown esophageal lesions in up to 40% of the patients.^25^

In an editorial we wrote in 2005 for the Journal of Cardiovascular Electrophysiology (JCE) regarding one of the measures to prevent AEF after the three initial cases were published in the literature, we warned: "Every time a new intervention technique is proposed, a new complication or adverse effect is expected to occur. It is just a matter of time! In that situation, our efforts have to be focused on how to identify, to minimize, to avoid it!".^26^ The perception is that this message remains current and that the physicians involved in the treatment of patients with AF should be informed and alert, because the occurrence of AEF still persists, despite the intense technological progress that has occurred in AF ablation during this period.

In conclusion, a better refinement of the technology is still necessary not only to make catheter AF ablation more effective but also safer. Electrophysiologists should be attentive to technical details that may prevent esophageal lesions, and the clinicians who follow up the patients must be alert to identify early eventual lesions that should be treated promptly.
